# A Novel Antibody-Toxin Conjugate to Treat Mantle Cell Lymphoma

**DOI:** 10.3389/fonc.2019.00258

**Published:** 2019-04-10

**Authors:** Gulam M. Rather, Siang-Yo Lin, Hongxia Lin, Zoltan Szekely, Joseph R. Bertino

**Affiliations:** ^1^Departments of Pharmacology and Medicine, Rutgers Cancer Institute of New Jersey, Rutgers, The State University of New Jersey, New Brunswick, NJ, United States; ^2^Department of Pharmaceutics, Ernest Mario School of Pharmacy, Rutgers, The State University of New Jersey, Piscataway, NJ, United States

**Keywords:** activated matriptase, antibody drug conjugate, monomethyl auristatin-E, mantle cell lymphoma, xenograft

## Abstract

Matriptase is a transmembrane serine protease, synthesized as an inactive single-chain zymogen on the endoplasmic reticulum and transported to the plasma membrane. Matriptase is activated in different epithelial and some B-cell malignancies and changes its conformation and activity is inhibited mainly by its endogenous inhibitor HAI-1. Activated matriptase plays a key role in tumor initiation as well as tumor progression, including invasiveness, and metastasis. To target the anti-mitotic toxin (monomethyl auristatin-E) to activated matriptase, a novel antibody to activated matriptase was conjugated with this toxin via a valine-citrulline-PABA linker. In a previous study, this antibody-toxin conjugate was found to be effective against triple negative breast cancer cell lines and xenografts, alone, or in combination with cisplatin (1). In this study, we examined the anti-tumor effect of the antibody toxin conjugate (ADC) against activated matriptase positive mantle cell lymphoma cell lines (JeKo-1, Maver, Mino, and Z138). This ADC was cytotoxic to these cell lines with IC_50_s between 5 and 14 μg/mL. The ADC also showed a dose dependent anti-tumor effect on the JeKo-1 xenograft in mice without toxicity.

## Introduction

Mantle Cell Lymphoma (MCL), represents 6- percent of all lymphoma cases, and currently the survival time is 4–5 years, shorter compared to other hematologic malignancies (2–4). MCL cells express CD20, aberrant expression of CD5, and due to a translocation t(11;14)(q13;q32), overexpression of cyclin-D1, encoded by the CCND1 gene located on chromosome 11, which mediates cell cycle progression through the G1 phase (5, 6). The currently used drugs to treat MCL patients include bortezomib, ibrutinib, rituximab, bendamustine, and combinations of these drugs.

Matriptase, a glycoprotein (80–90 kDa), is a member of type II transmembrane serine proteases. It is synthesized as a latent single-chain structure and with many regulatory mechanisms and functions (7, 8), and is activated through an auto-activation step resulting in a disulfide-linked-two-chain structure. Following activation, matriptase is rapidly inactivated by its endogenous inhibitor HAI-1. This activated matriptase-HAI-1 complex remains present in most epithelial carcinomas and some B-cell malignancies (9–11). Importantly, while matriptase is present in a latent form on epithelial cells and B-cells, activated matriptase expression is mainly restricted to the membranes of epithelial tumors, and some B-cell malignancies, in particular MCL (10–12).

Of importance, given the increase in reactive oxygen species (ROS) and the acidic environment of solid tumors (ROS), these environments activate the matriptase zymogen (13–21).

In this study we show that a novel anti-matriptase antibody toxin (Monomethyl auristatin-E, MMAE) conjugate potently inhibited growth of mantle cell lymphoma cell lines (JeKo-1, Maver, Mino and Z138) and caused significant growth inhibition of the JeKo-1 xenograft *in vivo*.

## Materials and Methods

### Animals

NOD/SCID/IL2 receptor gamma chain null (NOD/SCID/IL2rg^null^, NSG) mice were obtained from the Jackson Laboratory (Bar Harbor, ME).

### Materials

For cell culture, RPMI 1640, and fetal bovine serum were from Invitrogen (Fisher Scientific).

### Cell Culture

The MCL cells (JeKo-1, Mino, Maver, and Z138) were cultured in 1X RPMI Media 1,640 (Life Technologies) containing 10% fetal bovine serum (FBS) at 37°C and 5% carbon dioxide. All the cell lines were obtained from American Type Culture Collection (ATCC) and were checked for mycoplasma by MycoAlert™ mycoplasma detection kit (Lonza USA).

### Western Blotting

The MCL cells were scraped into a micro centrifuge tube from petri-dishes after 75% confluency. After centrifugation, cell pellets were lysed in lysis buffer (20 mM Tris, pH 7.4) containing 1% triton-X100, a commercial protease inhibitor cocktail (Roche) and 1 mM 5,5′-dithio-bis(2-nitrobenzoic acid) (DTNB). Since, DTNB interferes with the Bradford reagent (Bio-Rad Laboratories), equal volume of protein samples was resolved by 10% SDS-PAGE, without any boiling and under non-reducing sample buffer conditions and transferred onto a nitrocellulose membrane (Bio-Rad Laboratories). After blocking the membrane with 5% non-fat dry milk prepared in Tris buffered saline with 0.1% Tween-20 (TBST), the membrane was incubated with the desired primary antibody M69 at 4°C overnight. The membrane was washed thrice in TBST and then incubated for 2 h at room temperature with the appropriate peroxidase-conjugated secondary antibody. Bands were visualized using an enhanced chemiluminescence kit (Pierce). Anti-glyceraldehyde 3-phosphate dehydrogenase (GAPDH) (from Millipore) and was used as a control. Anti-HAI-1, anti-Vinculin and anti-mouse secondary antibody were from Santa Cruz Biotechnologies. Anti-mouse secondary antibody was used to probe the ADC (mouse antibody recognizing human activated matriptase) and also to probe GAPDH, HAI-1, and Vinculin which are mouse generated.

### Cytotoxicity Assay

Five thousand cells per well were plated in RPMI 1,640 media supplemented with 10% FBS. After overnight culture, media was removed and fresh media containing the ADC was added and incubated for different time periods. To assess cell viability, the MCL cell lines with or without drug treatment were collected and cell viability was determined using the Vi-CELL™ Series Cell Viability Analyzer (Beckman Coulter, Carlsbad, CA). The 50% inhibitory concentration (IC_50_; the drug concentration required to obtain 50% cell kill compared to control) was determined using the non-linear regression curve fit of the graphs drawn by GraphPad Prism 4 software (GraphPad Software Inc., CA). All experiments were performed in triplicate, and all experiments were repeated at least three times.

### Migration Assay

MCL (suspension cells) cells were treated with ADC (IC_50_) for 48 h and washed twice with IX PBS. The cells were then serum starved for 1.5 h in FBS-free RPMI at 37°C and 5% carbon dioxide in presence of ADC. Three hundred microliters of FBS-free RPMI (8 × 10^5^ cells) were added to the top chamber of a cell culture insert (24-well format) of eight-micron pore size (Corning). Cells were treated with ADC (IC_50_) throughout the experiment (means ADC is present in FBS-free media in inserts as well as in the lower well of that insert). Inserts had been previously transferred to wells containing 700 mL of RPMI (containing 10% FBS) with or without ADC. After 24 h of incubation at 37°C and 5% carbon dioxide, cells were collected from both insert chamber and lower well (of 24-well plate) and checked for viability using the Vi-CELL™ Series Cell Viability Analyzer (Beckman Coulter, Carlsbad, CA). The percent viable cells migrated toward FBS (in lower well) of total viable cells added in insert, were plotted against ADC treatment. Each experiment was done at-least three times and in four replicates.

### Animal Studies

The JeKo-1 cell line was used for anti-tumor studies. Cells (10 × 10^6)^ in 100 μL of PBS were injected subcutaneously into the right flank of 6-week-old NSG female mice. Once tumors were palpable, the mice were randomized to different groups. Mice were treated i.p. with the ADC, and treatment periods were indicated by arrows. Saline was used as a control treatment. Tumor size and body weights were measured twice a week and the tumor volume was calculated using the formula width^2^ × (length/2). Results are presented as mean ± SEM.

### Histologic Preparation and Immunohistochemistry Staining

Samples were fixed in 4% formalin and paraffin-embedded. Immunohistochemistry was performed on 4 μm sections with antibodies to Ki67 (Santa Cruz Biotechnologies, USA) and Cleaved caspase-3 (Cell Signaling Technology USA #9661). Sections were developed and stained with hematoxylin and eosin using standard methods. All histological preparations and immunostaining were conducted by the Rutgers Cancer Institute of New Jersey Biospecimen Repository and Histopathology Core.

### Statistical Analysis

Statistical analysis was performed using Prism software (GraphPad). In all cases, ANOVA followed by two-tailed, unpaired Student *t*-tests was performed to analyze statistical differences between groups. *P-*values of <0.05 were considered statistically significant.

### Antibody-Toxin Conjugate Preparation and Characterization

The anti-matriptase antibody (M69) was generated against purified activated matriptase-HAI complex from human milk as described by Lin et al. (22). Seattle Genetics' valine-citrulline-PABA linker technology was used for conjugation of a potent tubulin-inhibitor, monomethyl auristatin-E (MMAE) to the M69 antibody. The valine-citrulline dipeptide based linker has been shown to be stable in circulation but cleavable by cathepsin B in the lysosome to generate free drug (23). Copper free click chemistry is used to load the toxin in a stoichiometrically controlled manner to M69 antibody under very mild conditions. The technology involves conjugating the linker-toxin with the lysine side chains on the antibody surface. The conjugation procedure does not affect the disulfide bridges between cysteines of the antibody, thus maintaining the structure of the antibody without any loss of antibody activity by misfolding or dissociation of antibody chains. Analysis by mass spectrometry (HR-MALDI-TOF) showed an increase of 7,000 Da average M.W. corresponding to an average of 3.5 toxin (MMAE) molecules linked to each mAb molecule (1).

## Results

### *In vitro* Cytotoxicity of M69-MMAE (ADC) Against Mantle Cell Lymphoma (MCL) Cell Lines

**Figure 1:**
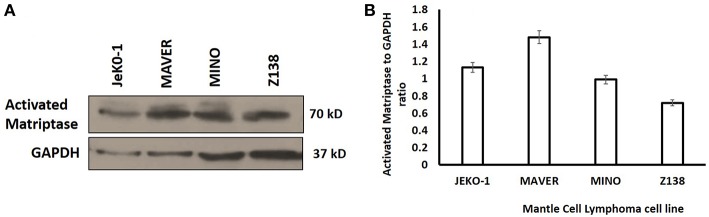
**(A)** Western Blot analysis of activated matriptase expression in Mantle Cell Lymphoma cells (JeKo-1, MAVER, MINO, and ZI38). Equal volume of lysate was loaded in 10% SDS-PAGE (see methods). **(B)** Activated matriptase to GAPDH ratio for all the four mantle cell lymphoma cell lines.

Activated matriptase expression was evaluated in different MCL cell lines (JeKo-1, Mino, Maver, and Z138) by Western blotting using the M-69 antibody that recognizes activated matriptase alone or in complex with HAI-1. The four cell lines showed increased levels of activated matriptase, although the level of expression varied (Figure 1). The expression level of hepatocyte growth factor activator inhibitor (HAI)-1 protein in mantle cells is shown in Figure S1.

**Figure 2:**
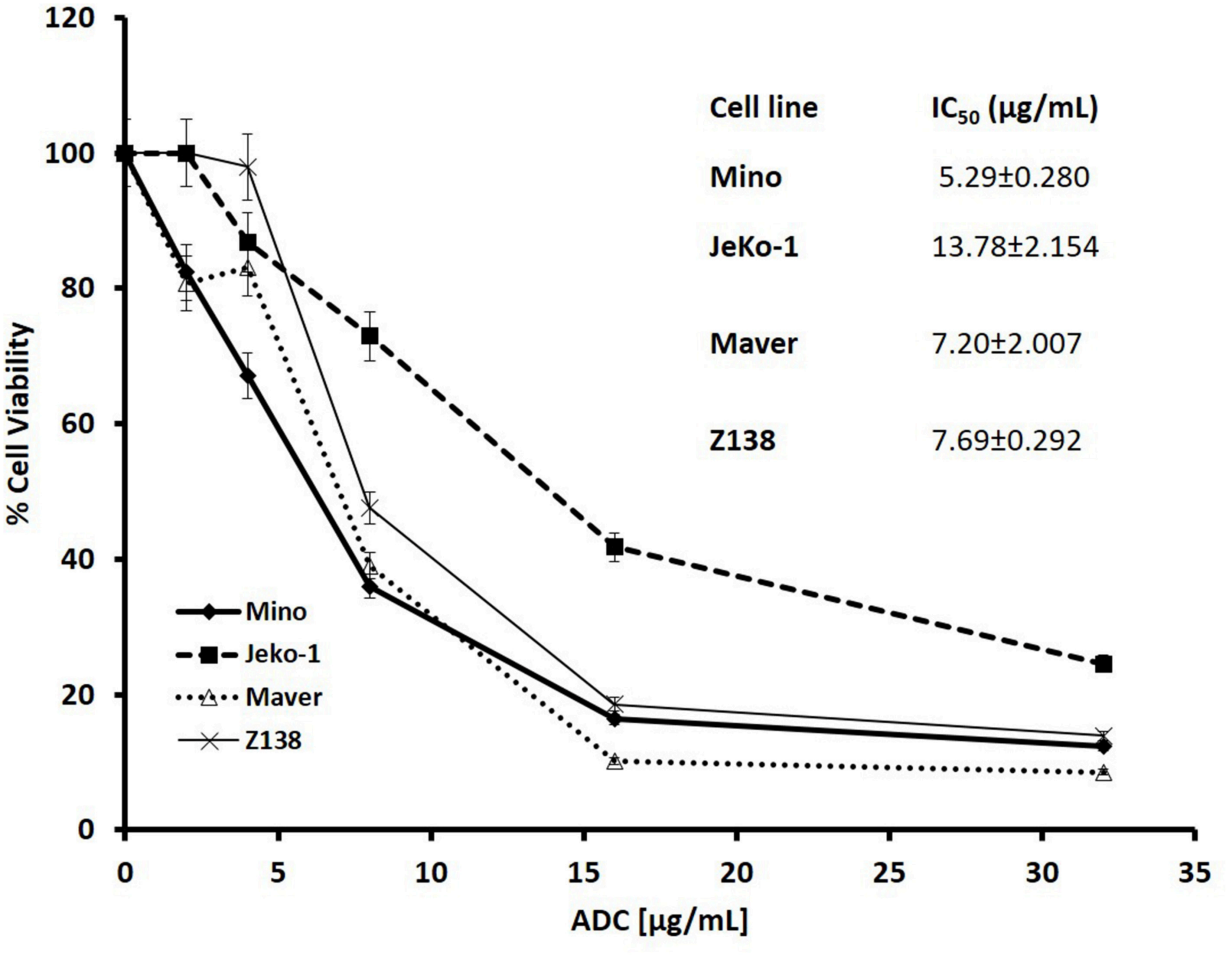
Cytotoxicity of M69-MMAE conjugate (ADC) against different MCL cell lines. Five thousand cells/well were plated in a 96-well plate and the cells were treated the next day with the ADC for 72 h. Cytotoxicity of the ADC was measured by trypan blue dye exclusion method using a Vi-Cell XR© cell viability analyzer (Beckman Coulter). All the reading points were carried out in triplicates. The IC_50_ values (insert) are calculated using GraphPad Prism 4 software. Results are presented as mean ± SEM.

**Figure 3:**
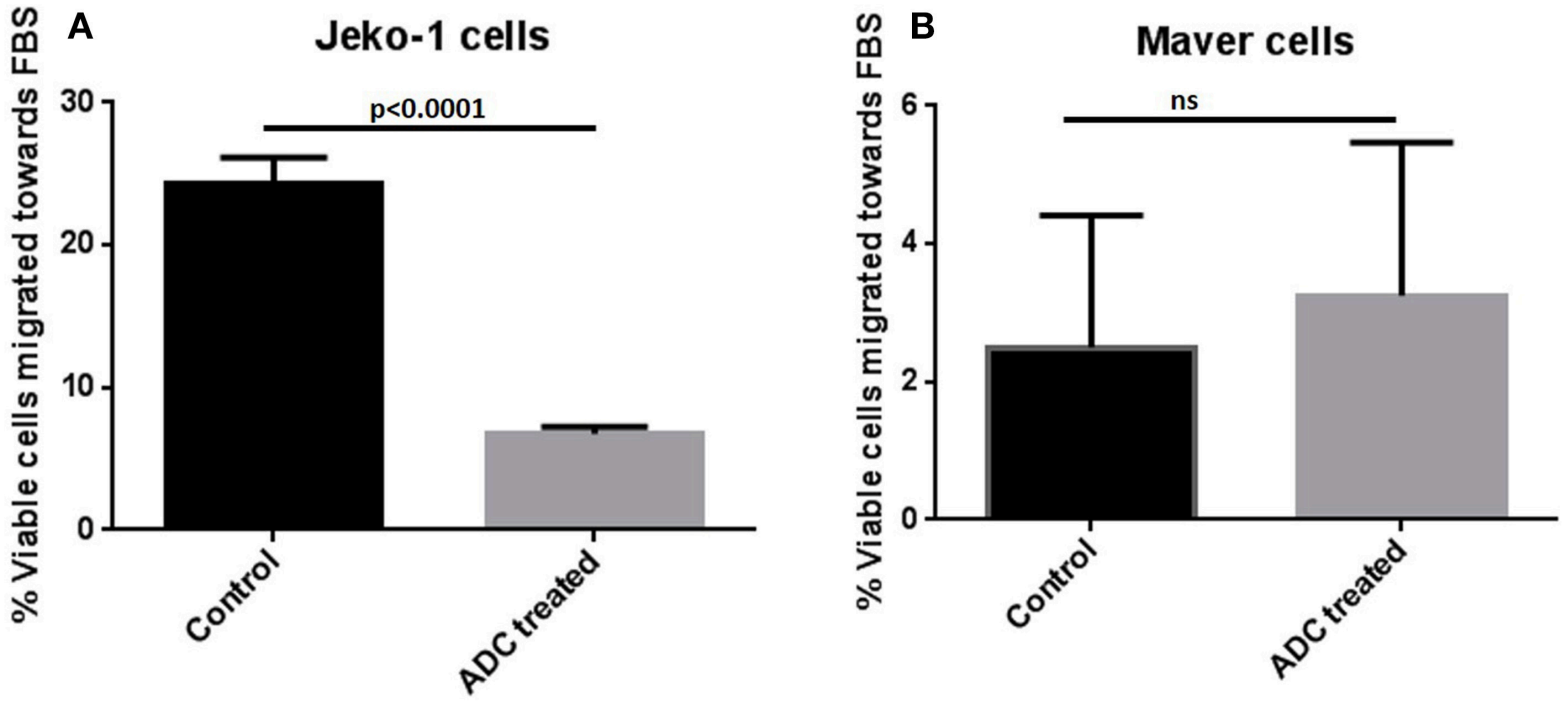
Effect of ADC on migration of MCL cell lines *in vitro*. **(A)** JeKo-1 **(B)** Maver cells. Cells were treated with ADC for 48 h and washed twice with 1X PBS and starved for 1.5 h in FBS-free RPMI and then added in a cell culture insert having 8-micron pore size in 300 μl of FBS-free RPMI (with and without ADC). The insert was transferred to a well containing 700 μl of FBS-containing RPMI (with and without ADC) for 24 h at 37°C and 5% carbon dioxide. Cells were checked for viability from both insert and lower well using the Vi-CELL™ Series Cell Viability Analyzer (Beckman Coulter, Carlsbad, CA). The percent viable cells migrated toward the lower well (having FBS-RPMI) of total viable cells added in insert were plotted against ADC treatment. Each experiment was done at-least three times and in four replicates. Results are presented as mean ± SEM.

Cytotoxicity studies showed that the ADC decreased the viability of all the cell lines (Figure 2) with IC_50_s at single digit μg/ml of the conjugate. As 3.5 molecules of toxin are bound on the average to each antibody molecule, the IC_50_ values for the toxin ranged from 125 to 611 pM. Based on the IC_50_ values, Mino, Maver and Z138 cells were 1.8–2.6-fold more sensitive to ADC compared to JeKo-1. In order to check whether the ADC is stable in media, the ADC was incubated (37°C and 5% carbon dioxide) in complete media (RPMI with 10% FBS) for 48 h before used for cytotoxicity test and it was found that 48 h incubated ADC and fresh ADC are equally effective against Maver cell line as shown in Figure S2. In order to study the role of matriptase in metastasis and invasiveness, the ADC was found to inhibit the migration of JeKo-1 cells *in vitro*. Of interest, only a small percent of cell from the Maver cell line migrated as compared to the JeKo-1 cell line, and the ADC did not enhance migration (Figure 3).

### JeKo-1 Xenograft Studies

**Figure 4:**
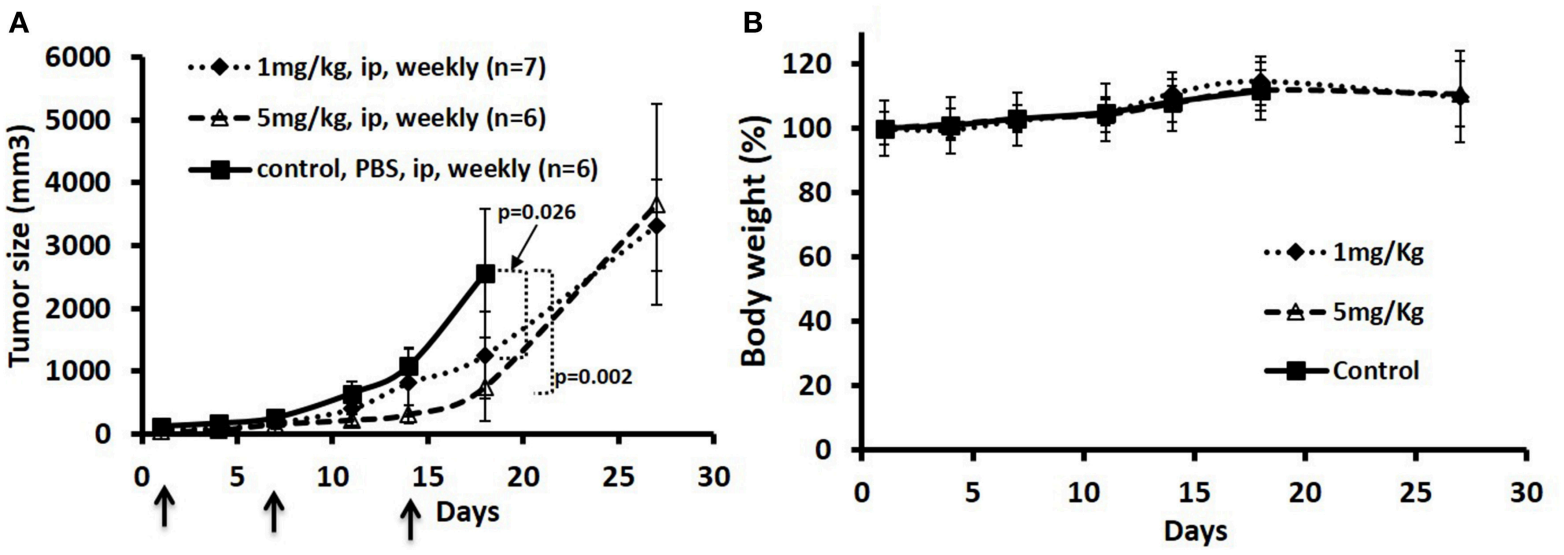
Treatment of JeKo-1 xenografts in mice using different doses of matriptase-MMAE conjugate (ADC). **(A)** Xenograft studies with M69-MMAE. NOD/SCID mice were inoculated with 10 × 10^6^ JeKo-1 cells in PBS in the right flanks. When the tumor was palpable (100–200 mm^3^), mice (*n* = 19) were randomized into: control (antibody alone), 1 and 5 mg/kg M69-MMAE treatment groups. M69-MMAE was administrated by i.p. weekly x 2. Tumor volume was measured twice a week. Tumor volumes were calculated using the formula width^2^ x (length/2). Results are presented as mean ± SEM **(B)** Mice body weight change in the control and treatment groups. Treatments are shown by arrows.

To test the anti-tumor effects of the ADC in one of the MCL tumors in a mouse model, we elected to test the JeKo-1 cell line. We tested two dose schedules of the ADC: 1 vs. 5 mg/Kg administered i.p. weekly. The 5 mg/Kg weekly dose was more effective than the 1 mg/Kg dose. Even at the higher dose, there were no signs of toxicity as measured by observation and weight loss (Figure 4). Previous studies with the naked antibody showed that it had no anti-tumor activity *per se* (1).

**Figure 5:**
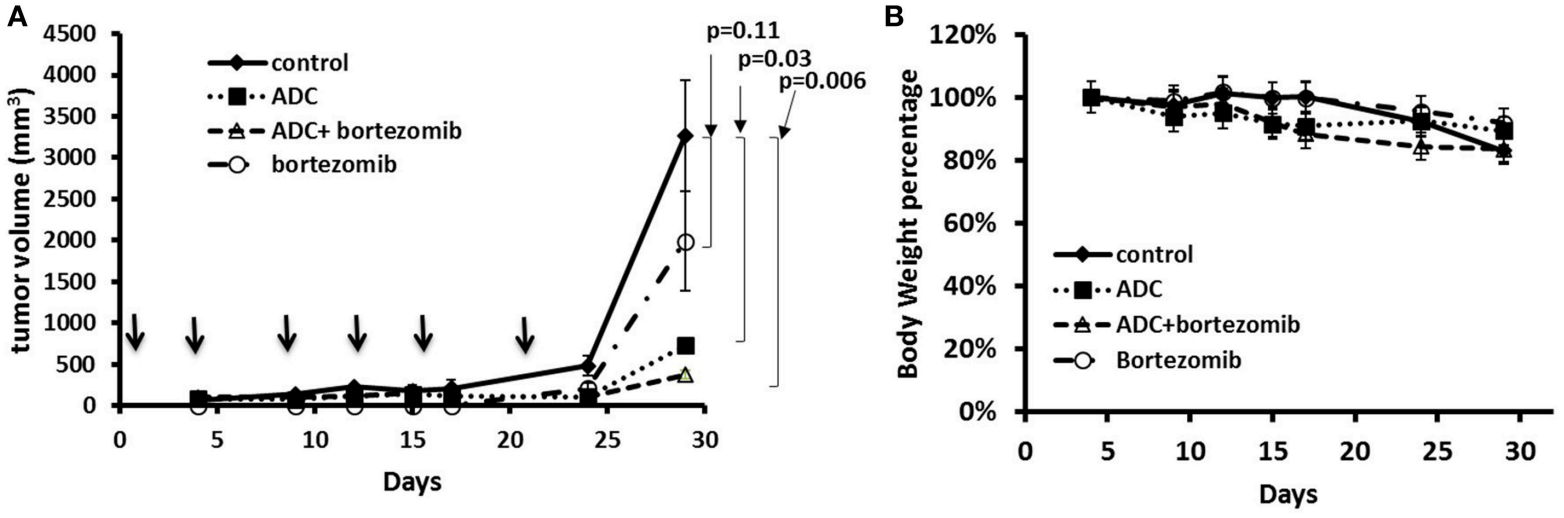
Treatment of JeKo-1 xenografts in mice using the matriptase-MMAE conjugate (ADC) and bortezomib. **(A)** Xenograft studies with M69-MMAE and bortezomib. NOD/SCID mice were inoculated with 10 × 106 JeKo-1 cells in PBS in the right flank. When tumors were palpable, mice were randomized into control, bortezomib, M69-MMAE, and bortezomib plus M69-MMAE treatment groups. M69-MMAE (5 mg/kg) was administrated by i.p. twice weekly for 3 weeks. Bortezomib (0.75 mg/kg) was given i.p. weekly. Bortezomib and M69-MMAE were given together with the same dose schedule. Tumor volume was measured twice a week, and the tumor volume calculated using the formula width^2^ x (length/2). Results are presented as mean ± SEM. **(B)** Mice body weight percentage during the treatment. Treatments are shown by arrows.

As bortezomib is used to treat MCL, alone and in combination, we also tested the ADC in combination with bortezomib in a JeKo-1 xenograft study. Using a similar inoculum, this tumor grows rapidly in NOD-SCID-gamma mice, and the biweekly 5 mg/kg dose schedule, both bortezomib and the ADC caused marked tumor growth inhibition (*p* = 0.006). The combination of bortezomib and the ADC was more effective than either drug alone (Figure 5).

**Figure 6:**
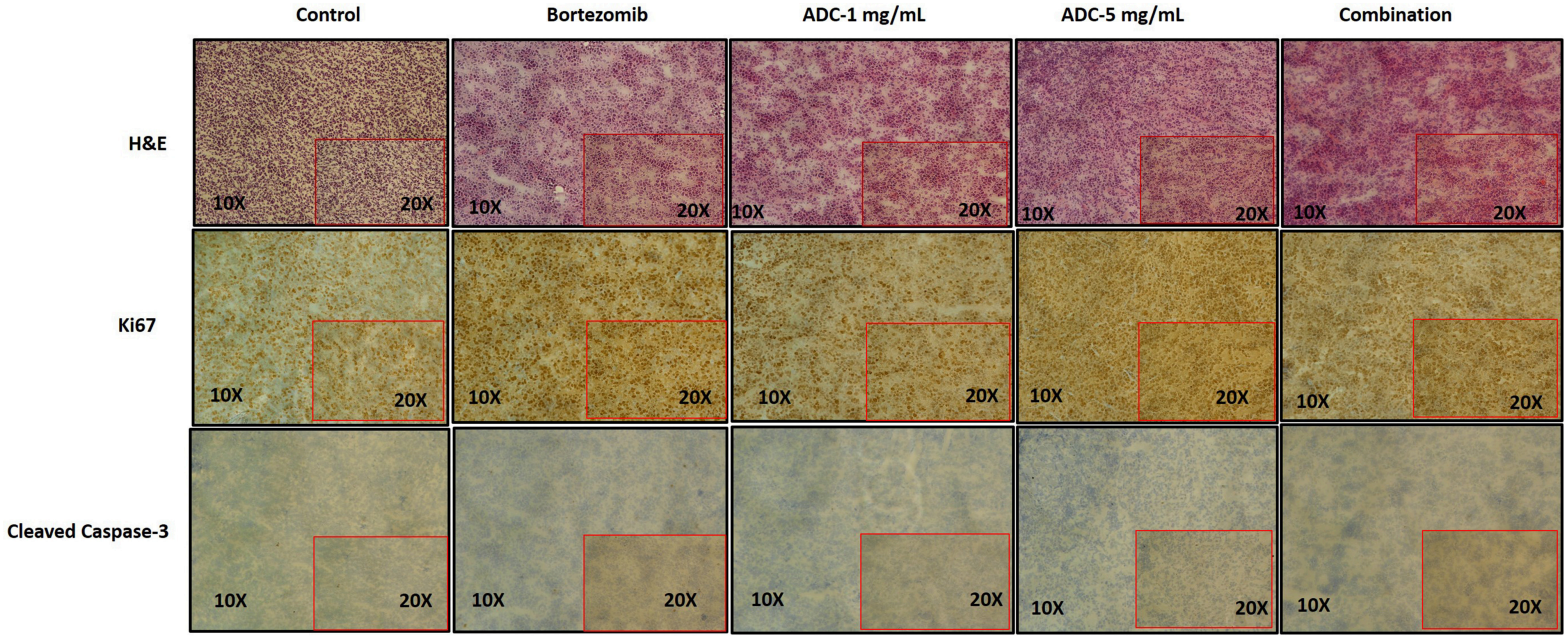
Immunohistochemistry staining of JeKo-1 tumors extracted from mice in Figures 4, 5 against various biomarkers. The tumors were harvested from control, 1 and 5 mg/kg (i.p. weekly) M69-MMAE treatment groups from experiment four and Bortezomib and M69-MMAE combination (Bortezomib (0.75 mg/kg, i.p. weekly and M69-MMAE, 5mg/kg i.p. twice weekly) from experiment five. Ki67 staining showing proliferation of JeKo-1; and Cleaved caspase-3 showing the apoptotic cell death. The tonsil tissue was used as a positive control for various IHC staining.

We harvested the tumors at the end of the experiment and then used immunohistochemistry to test for various biomarkers. Figure 6 showed that there was no significant change in Ki-67 staining; however, cleaved caspase-3 staining (apoptosis) showed a significant increase in the combination group (ADC with bortezomib) compared to either drug alone.

## Discussion

Brentuximab vedotin (Adcetris), consisting of an antibody that targets CD30, conjugated with MMAE is approved for the treatment of Hodgkin disease, as well anaplastic large cell lymphoma (ALCL) (24). CAT-3888 (BL22), another immunotoxin, which targets the CD22 antigen on certain lymphoma cells, attached to a bacterial Pseudomonas exotoxin, PE38, has shown activity against hairy cell leukemia (HCL) in early clinical trials (25). CAT-8015 (moxetumomab pasudotox), an updated version of this drug, is now being studied for use against lymphomas (26).

Our novel antibody against activated matriptase, overexpressed in B-cell lymphoma and epithelial tumors and involved in tumorogenesis, invasiveness and metastasis (27–29), conjugated with the tubulin binding, mitotic inhibitor toxin, monomethyl auristatin E (MMAE), demonstrates that activated matriptase is a bonafide target for use with antibodies that recognize activated matriptase, armed with a toxin. The pre-incubated ADC was as potent as the non-incubated fresh ADC, indicating that the ADC was stable in FBS and media. The *in vitro* experiments also confirmed that this ADC showed significant inhibition of migration of JeKo-1 cells. No observable toxicity was found with this ADC, however, as this is a mouse antibody that recognizes human, but not mouse matriptase, other toxic effects of the ADC would not be noted. We currently have constructed a chimeric matriptase antibody, suitable for toxicity studies in a primate model and for Phase I trials in humans.

Future plans are to use this ADC alone and in combination with other chemotherapeutic drugs (bortezomib and ibrutinib) in primary MCL xenografts with the goal of generating additional sufficient preclinical data to allow for future clinical development.

## Ethics Statement

All the cell line studies were performed through Rutgers Cancer Institute of New Jersey using protocols approved by the Rutgers Environmental Health and Safety (REHS). Animal experiments were conducted in accordance with Rutgers Cancer Institute of New Jersey Animal Care and Use Committee guidelines using an approved protocol number 15-040.

## Author Contributions

GR, S-YL, HL, ZS, and JRB conception and design, development of methodology, analysis and interpretation of data (e.g., statistical analysis, biostatistics, computational analysis), writing, review, and/or revision of the manuscript, and administrative, technical, or material support (i.e., reporting or organizing data, constructing databases). GR, S-YL, and HL acquisition of data (provided animals, acquired and managed patients, provided facilities, etc.). S-YL, ZS (for ADC conjugation study), and JRB (overall) study supervision.

### Conflict of Interest Statement

S-YL and JRB are founders of Xiconic, LLC. ZS is an officer of Xiconic, LLC. The remaining authors declare that the research was conducted in the absence of any commercial or financial relationships that could be construed as a potential conflict of interest.

## Supplementary Material

Figure S1 Western Blot analysis showing the hepatocyte growth factor activator inhibitor (HAI)-1 expression in Mantle Cell Lymphoma cells (Lane1: JEKO-1, Lane 2: MAVER). 10% SDS-PAGE was used.

Figure S2 Cytotoxicity of M69-MMAE conjugate (ADC) after 48 h incubation in complete media against Maver cell line. Five thousand cells/well were plated in a 96-well plate and the cells were treated the next day with the ADC (fresh and 48 h incubated one) for 72 h. Cytotoxicity was measured using an MTS assay. All the reading points were carried out in triplicates. Results are presented as mean ± SEM.
