# Supplementary figures and images for: A Novel Antibody-Toxin Conjugate to Treat Mantle Cell Lymphoma
Source: Front Oncol. 2019 Apr 10;9:258. doi: 10.3389/fonc.2019.00258 (PMC6467949; doi:10.3389/fonc.2019.00258)

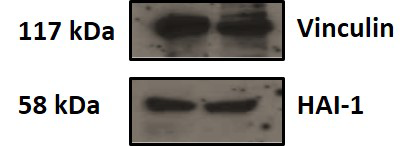

Supplement: Figure S1 — Western Blot analysis showing the hepatocyte growth factor activator inhibitor (HAI)-1 expression in Mantle Cell Lymphoma cells (Lane1: JEKO-1, Lane 2: MAVER). 10% SDS-PAGE was used. [file Image_1.TIF]

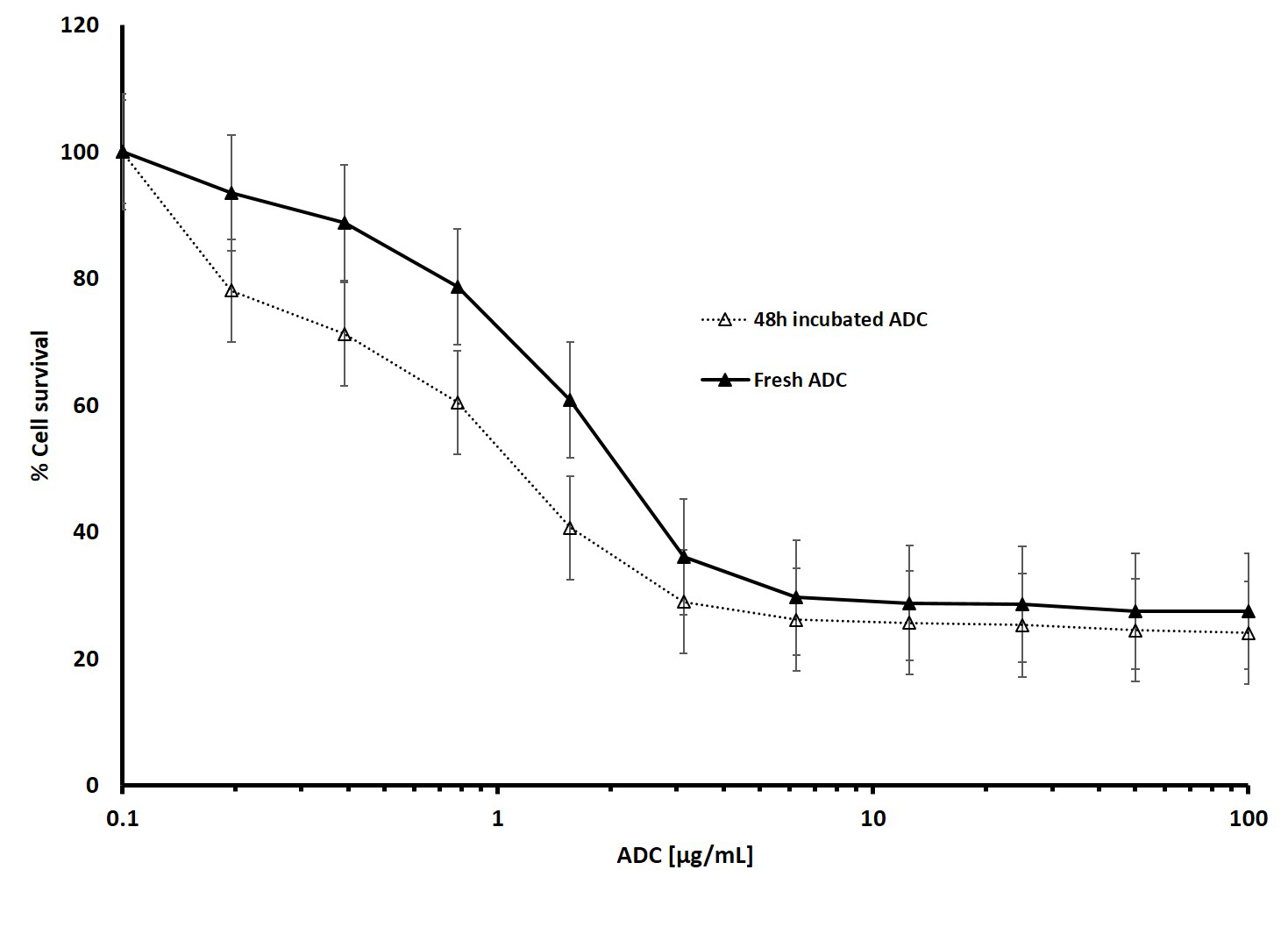

Supplement: Figure S2 — Cytotoxicity of M69-MMAE conjugate (ADC) after 48 h incubation in complete media against Maver cell line. Five thousand cells/well were plated in a 96-well plate and the cells were treated the next day with the ADC (fresh and 48 h incubated one) for 72 h. Cytotoxicity was measured using an MTS assay. All the reading points were carried out in triplicates. Results are presented as mean ± SEM. [file Image_2.TIF]
